# Correction: *Mycobacterium ulcerans* culture results according to duration of prior antibiotic treatment: A cohort study

**DOI:** 10.1371/journal.pone.0334452

**Published:** 2025-10-13

**Authors:** Brodie Tweeddale, Fiona Collier, Nilakshi T. Waidyatillake, Eugene Athan, Daniel P. O’Brien

The first author’s name is spelled incorrectly. The correct name is: Brodie Tweeddale. The correct citation is: Tweeddale B, Collier F, Waidyatillake NT, Athan E, O’Brien DP (2023) *Mycobacterium ulcerans* culture results according to duration of prior antibiotic treatment: A cohort study. PLoS ONE 18(4): e0284201. https://doi.org/10.1371/journal.pone.0284201

## References

[1] Tweedale B, Collier F, Waidyatillake NT, Athan E, O’Brien DP. *Mycobacterium ulcerans* culture results according to duration of prior antibiotic treatment: A cohort study. PLoS One. 2023;18(4):e0284201. doi: 10.1371/journal.pone.0284201 37093836 PMC10124831

