# Supplementary material for: Mycobacterium ulcerans culture results according to duration of prior antibiotic treatment: A cohort study
Source: PLoS One. 2023 Apr 24;18(4):e0284201. doi: 10.1371/journal.pone.0284201 (PMC10124831; doi:10.1371/journal.pone.0284201)
Supplement: Attachment — Submitted filename: Comments.docx [file pone.0284201.s001.docx]

Comments

Summary: the objective of the study is to investigate the length of antibiotic treatment duration required to achieve sterilization of *M.ulcerans* disease lesions. To reach such a target, the authors studied a cohort of 92 patients diagnosed with *M.ulcerans* disease who, from May 25, 1998 to June 30, 2019, had antibiotic treatment (mainly daily rifampicin + clarithromycin or ciprofloxacin) before surgery at Barton Health, Victoria, Australia and had culture of the excision lesions on Loewenstein Jensen medium.

Comment to the authors

The manuscript is well presented but, while the results of study are totally based on the sterilization or not of the excised lesions, almost no information is given on the technical means of the culture of excised specimens. It is only written “Mycobacterial cultures from surgical specimens were performed at Barwon Health microbiological laboratories using Lowenstein-Jensen media and incubated for 12 weeks (page 5, lines 129-130)”. More details are needed: time between lesion excision surgery and culture, temperature to which the excised lesions are kept, the interval of time between surgical excision and culture, mincing of the specimen, decontamination procedures if any, dilutions of the excision specimen, amount seeded on Loewenstein Jensen medium and culture temperature. All precisions on the culture procedures are decisive for the results interpretation. They are more important and far more decisive than all statistical and clinical details.

Page 6, lines 144-155 are Greek for the reviewer. Is it possible to make them simpler when the only important factor for interpretation of data and treatment recommendations is bacteriology and not statistics or clinics (5 pages on statistics and clinics versus two lines on bacteriology!)?

Page 11 line 210. The word “sterilized” is not appropriate. I suggest the change of wording. Negative culture of excision specimen is not at all synonymous of sterilization (as pointed out by the authors on page 13, line 260)
