# Supplementary material for: Mycobacterium ulcerans culture results according to duration of prior antibiotic treatment: A cohort study
Source: PLoS One. 2023 Apr 24;18(4):e0284201. doi: 10.1371/journal.pone.0284201 (PMC10124831; doi:10.1371/journal.pone.0284201)
Supplement: Attachment — Submitted filename: M ulcerans review.docx [file pone.0284201.s002.docx]

**M. ulcerans review:**

This paper describes the results of a 20-year prospective observational cohort relating to antibiotic duration prior to surgery for M. ulcerans diseases in Victoria, Australia, adding further evidence that shorter antimicrobial durations appear to be effective at sterilizing M. ulcerans disease lesions (under the 8 weeks recommendation). This paper is well-written and clear and contributes the literature on BU and its treatment. The paper, at times, lacks a few details, but I suspect that these may be corrected quite easily during revision. Specifically, the paper is limited by the absence of clinical outcomes, the lack of a more detailed description of the limited sensitivity of M. ulcerans culture and does not discuss the clinical and pathobiological diversity of BU between Australia and Africa. All told, I believe this paper should be published in PLOS ONE after the following revisions are addressed.

Suggested clarifications:

If possible, please describe when antimicrobial regimen changed, as only initial antimicrobial regimen was included.

Please include a mention of the sensitivity of culture in the introduction.

Please describe WHO classification of BU in the introduction as referred to multiple times in text. Consider mentioning that a minority of WHO I lesions may heal spontaneously.

Clarify number of WHO III with associated osteomyelitis.

Describe level of surgical debridement and if skin grafting occurred.

If possible, please comment on secondary infections?

I suspect that BCG is not administered in Victoria, Australia. As this is relevant to BU manifestation, please indicate whether this population received the BCG vaccine.

If possible, add whether paradoxical deterioration occurred during antimicrobial therapy.

The age discrepancy between BU in Australia (60s) and Africa (5-15) is interesting. Please consider commenting on why BU is commonly seen in children in West Africa and older adults in Australia? Differences in water-related exposure/ behaviour? Mosquito-exposure?

Please comment on the differences in Mycolactone A and B (West Africa) and C (Australia) in different geographic areas and possible differences in virulence, limiting global extrapolation of the findings.

Small comments:

Line 57: please add “accent circumflex” to the o in Côte d’ivoire and accent aigu to Bénin.

Line 59: If possible, please provide numerical data regarding the increase in BU in South_eastern Australia.

Line 68: Please name the adverse drug effects. Hepatoxocity due to rifampin? Other drug effects?

Line 70: If possible, please provide duration of follow-up for this study. Eg: clinical cure and no relapse after X months of follow-up.

Line 85: 100% cure in how many patients? Please list sample size in this study.

Lines 173: please describe duration of follow-up for establishing that “no disease relapses” occurred.

Please describe most common other regimen 5%.
